# Extracellular vesicles protect glucuronidase model enzymes during freeze-drying

**DOI:** 10.1038/s41598-018-30786-y

**Published:** 2018-08-17

**Authors:** Julia Frank, Maximilian Richter, Chiara de Rossi, Claus-Michael Lehr, Kathrin Fuhrmann, Gregor Fuhrmann

**Affiliations:** 1 Helmholtz Institute for Pharmaceutical Research Saarland (HIPS), Helmholtz Centre for Infection Research (HZI), Biogenic Nanotherapeutics group (BION), Campus E8.1, 66123 Saarbrücken, Germany; 2 Helmholtz Institute for Pharmaceutical Research Saarland (HIPS), Helmholtz Centre for Infection Research (HZI), Department of Drug Delivery (DDEL), Campus E8.1, 66123 Saarbrücken, Germany; 3 Department of Pharmacy, Saarland University, Campus Building E8.1, 66123 Saarbrücken, Germany

**Keywords:** Nanobiotechnology, Nanoparticles, Diagnostic markers

## Abstract

Extracellular vesicles (EVs) are natural nanoparticles that play important roles in intercellular communication and are increasingly studied for biosignalling, pathogenesis and therapy. Nevertheless, little is known about optimal conditions for their transfer and storage, and the potential impact on preserving EV-loaded cargoes. We present the first comprehensive stability assessment of different widely available types of EVs during various storage conditions including −80 °C, 4 °C, room temperature, and freeze-drying (lyophilisation). Lyophilisation of EVs would allow easy handling at room temperature and thus significantly enhance their expanded investigation. A model enzyme, β-glucuronidase, was loaded into different types of EVs derived from mesenchymal stem cells, endothelial cells and cancer cells. Using asymmetric flow field-flow fractionation we proved that the model enzyme is indeed stably encapsulated into EVs. When assessing enzyme activity as indicator for EV stability, and in comparison to liposomes, we show that EVs are intrinsically stable during lyophilisation, an effect further enhanced by cryoprotectants. Our findings provide new insight for exploring lyophilisation as a novel storage modality and we create an important basis for standardised and advanced EV applications in biomedical research.

## Introduction

Specific cellular transfer of biological and therapeutic molecules is a key step for many physiological and biomedical processes. In recent years, extracellular vesicles (EVs) have gained substantial interest as such effective conveyers of intercellular communication^1,2^. EVs are cell-derived nanoparticles that are increasingly recognised as important mediators of biosignals during physiological and pathological processes^3,4^. They have recently raised excitement for the development of novel diagnostics^5^, drug therapies for cancer^6^, infection research^7^ and anti-inflammatory treatment^8^, and for other advanced engineering avenues^9–11^. EVs possess potential advantages over synthetic nanoparticles for biomedical applications, including physiological composition, reduced immune recognition and natural targeting abilities^12,13^. Although some EV applications have already reached clinical evaluations^2^, a major challenge for their extended utilisation in both fundamental biological and applied medical research is to establish stable and reproducible storage conditions without compromising their biological functionality^14^, with nucleic acid and protein composition holding an important role^15^. Recent efforts on harmonisation and centralised knowledge of EV characterisation have contributed to improved understanding of EV handling^16^ and the international consensus recommends their storage at −80 °C^17^, but it was also shown that frozen storage may alter the biological activity of EVs^18^. Overall, it remains scientifically debated whether individual manipulation of EVs under different conditions may change their physical and physiological properties.

Lyophilisation (freeze-dying) of EVs would allow easy handling at room temperature (RT) and thus significantly boost their expanded investigation but detailed scientific evaluation of this condition is lacking to date. It was recently shown that EVs are stable upon freezing and that their selective biological interaction with target cells is not compromised when sugar stabilisers were used^19^. Although important, this study employed only one type of EV-like particles which were not isolated from a common cell line (*i*.*e*., murine pancreatic beta cell line MIN6), and only freezing conditions with a single type of cryoprotecting agent (*i*.*e*., trehalose) were assessed. Besides, EV lyophilisation has only been investigated in patents (US14/958804) using specific cell sources (*i*.*e*., human cardiosphere-derived cells) that are not broadly available to the research community. Moreover, academic conclusions drawn from such studies are often rather low which motivated us to perform an extensive investigation of EV stability and functionality upon lyophilisation and other conditions. We comprehensively evaluated the stability of various widely available types of EVs, such as mesenchymal stem cells, endothelial cells and cancer cells, in comparison with liposomes as synthetic control nanoparticles^20^. Upon glucuronidase model enzyme loading we assessed the bioactivity of EVs and liposomes under different settings, including −80 °C, 4 °C, room temperature (RT) and lyophilisation. Asymmetric flow field-flow fractionation (AF4) was applied to assess the amount of enzyme stably encapsulated into EVs before and after lyophilisation. We moreover studied the effect of different cryoprotecting agents to preserve EV functionality during lyophilisation and compared their ability to counterbalance enzyme degradation. Our findings encourage exploring lyophilisation as EV storage alternative and we create a fundamental basis for standardised EV applications in biomedical research.

## Results and Discussion

**Figure 1:**
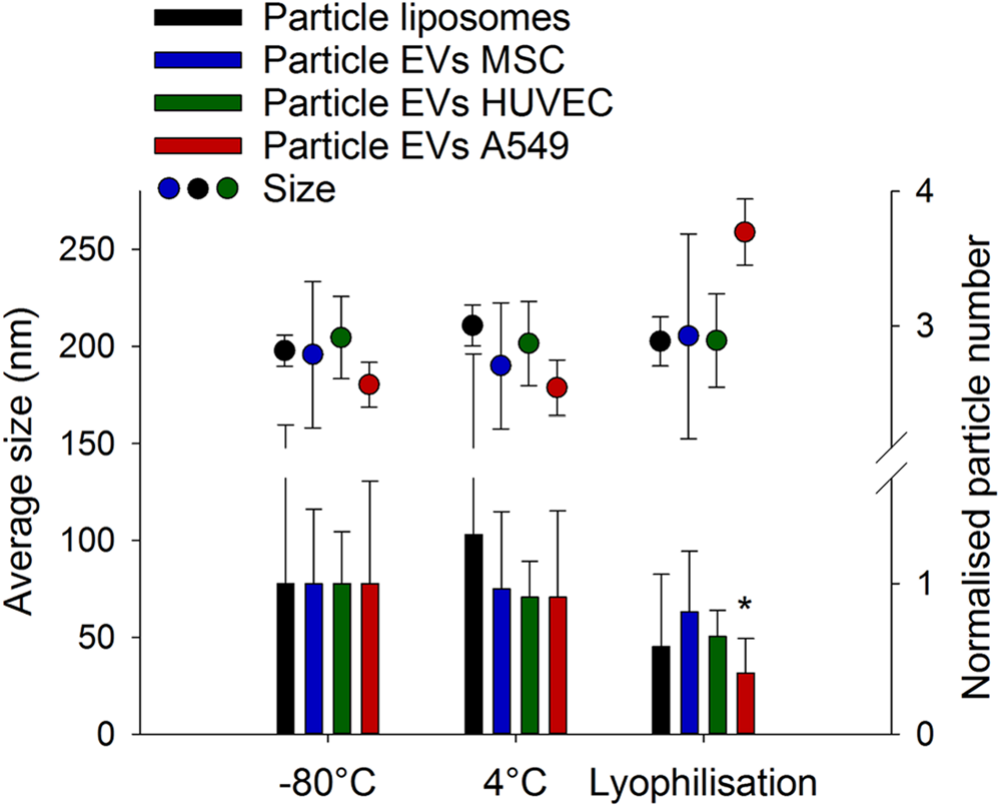
Impact of lyophilisation on native EVs. Unloaded EVs from MSC, HUVEC and A549 cells, and liposomes were assessed concerning average size and particle concentration during short term storage of 2 d at −80 °C, 4 °C or after lyophilisation. For each experiment, the particle concentration was normalised to the average particle concentration at −80 °C. Mean ± SD, *n* = 4–9, *p < 0.05 *vs*. −80 °C (ANOVA followed by Dunnett *post-hoc* test).

We selected a broad spectrum of cell sources including human mesenchymal stem cells (MSC), human umbilical vein endothelial cells (HUVEC) and human A549 lung cancer cells (A549) due to their wide-ranging importance in regenerative medicine applications^21^, drug delivery^22^ and cancer pathophysiology^23^. Cells were kept under serum-free conditions (SI Fig. 1) and their EVs were isolated from cell culture supernatants by differential ultracentrifugation followed by size exclusion chromatography^22^. Ultracentrifugation in combination with size exclusion chromatography as it was applied here is one of the most commonly accepted methods to isolate EVs and purify them from potential co-isolates^24,25^. Other isolation techniques include precipitation, filtration or magnetic pull-down but are mostly used for larger samples sizes^25^. EVs were stored in their native state at −80 °C, 4 °C, at RT or upon direct lyophilisation without any additives. For all experiments, liposomes composed of 1,2-dimyristoyl-*sn*-glycero-3-phosphocholine (DMPC) and 1,2-dipalmitoyl-*sn*-glycero-3-phosphocholine (DPPC) (DMPC:DPPC = 2:3) were used as synthetic comparator for EVs. As seen from Fig. 1, EVs showed an average initial size of around 200 nm and there was no immediate effect on size during freeze-drying compared to storage at −80 °C and compared to plain liposomes. EV concentrations decreased during lyophilisation with significant effects for A549 EVs only. No change in particle number was observed for MSC EVs indicating their inherent stability as also reported for bacteria-derived vesicles used for vaccine applications^26^. Interestingly, liposome samples showed very large variations in their particle number, both at 4 °C and at −80 °C but no statistical differences were detected.

**Figure 2:**
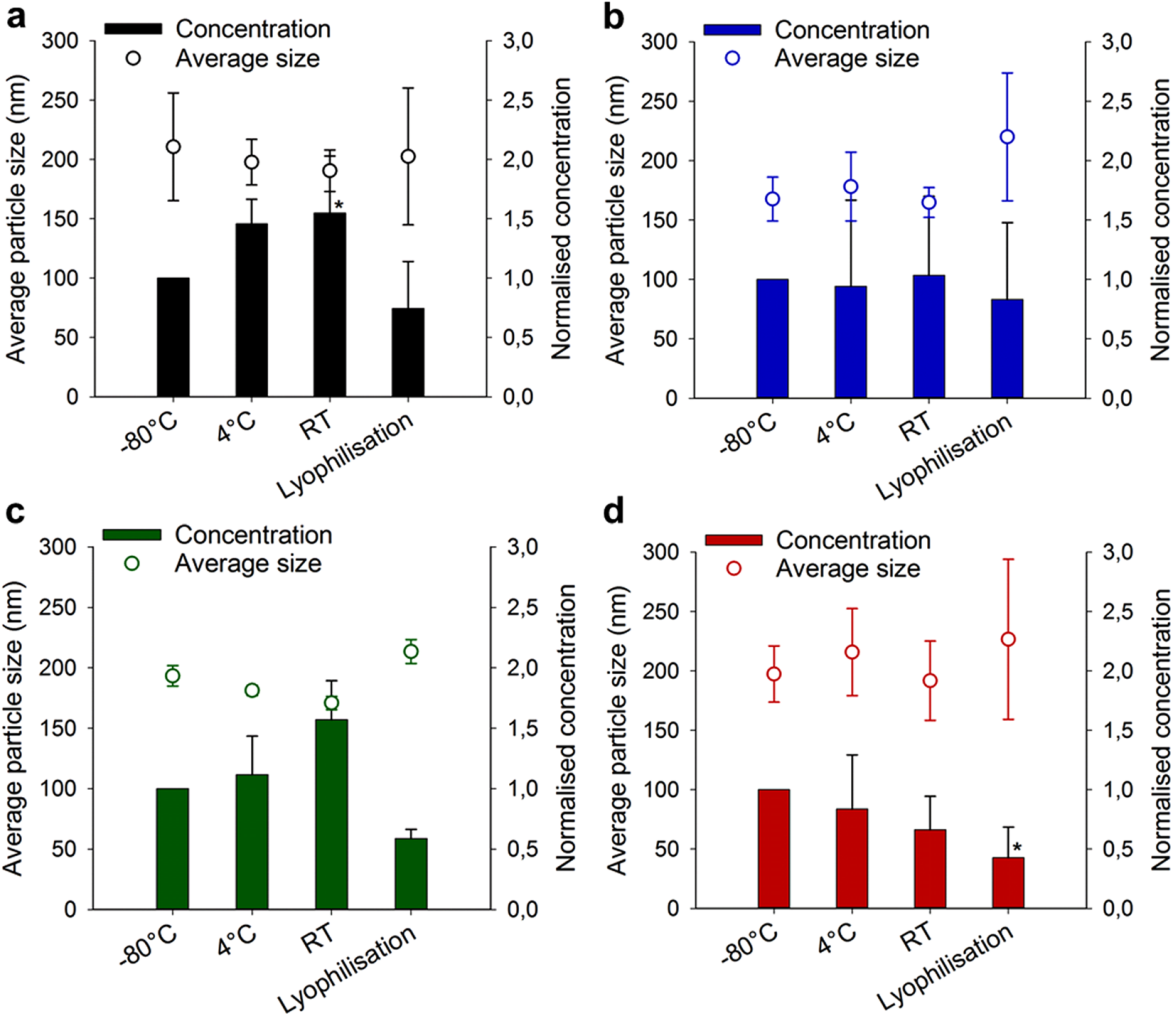
Long-term storage stability of enzyme-loaded EVs. Average size and particle concentration of (**a**) liposomes, and EVs from (**b**) MSC, (**c**) HUVEC and (**d**) A549 cells after loading of model enzyme glucuronidase was assessed. Natural and synthetic vesicles were stored for 14 d at various conditions (−80 °C, 4 °C, RT, and lyophilisation) and analysed by nanoparticle tracking analysis. Mean ± SD, *n* = 4–7, *p < 0.05 *vs*. −80 °C (ANOVA followed by Dunnett *post-hoc* test *versus* control samples −80 °C).

Based on these findings we loaded EVs with the enzyme glucuronidase to provide a functional biological readout using the substrate fluorescein β-D-glucuronide that is cleaved by glucuronidase releasing fluorescein. Enzymes are ideal as macromolecular model entities due to their inherent susceptibility to physiological conditions^27^ and are thus suitable for assessing whether encapsulation into EVs can protect these sensitive biomolecules under different conditions. Glucuronidase was encapsulated into EVs by saponin pre-treatment as reported previously^22^. This mild loading method did not induce changes in EV size compared to the unloaded vesicles (SI Fig. 2). Surprisingly, we observed an increase in size span for liposomes (SI Fig. 2a), indicating that the natural EVs may have a better stability upon loading using the saponin method. Both liposomes and EVs were purified by size-exclusion chromatography (SEC) (SI Fig. 3) and we have shown previously that they have comparable enzymatic activity^28^. SEC is known to be efficient for purification of EVs from both protein aggregates and non-encapsulated free drugs^22,29,30^. Concentrations of 1–1.5 × 10^10^ vesicles/mL were lyophilised without additives, and stored at −80 °C, 4 °C or RT for 14 days. Figure 2 illustrates the evolution of size and particle concentration of EVs compared to plain liposomes (Fig. 2a). We observed a reduction in EV and liposome number for all storage conditions compared to the native vesicles at day 0 (SI Fig. 4), but when comparing −80 °C, 4 °C, RT or lyophilisation among each other MSC EVs did not exhibit any fundamental change in yield (Fig. 2b). Lyophilisation appeared to induce a small but not significant increase in particle size. These results further underline an inherent stability of MSC EVs and the suitability of lyophilisation as valid storage alternative for these therapeutically important EVs. For HUVEC EVs a trend towards reduced particle number upon lyophilisation was observed but this effect was not significant due to overall variations in the data (Fig. 2c). Interestingly, HUVEC EVs showed the smallest standard deviation for their size determination, an effect already seen at short term storage. EVs from cancer A549 cells showed the least stability upon different storage conditions as indicated by the decreasing trend in particle concentration from −80 °C, to 4 °C, RT and lyophilisation (Fig. 2d). Although the size of A549 EVs was not substantially altered during lyophilisation, direct freeze-drying of these particles without any additives could not be recommended during biological studies. Interestingly, liposome storage at 4 °C or RT induced slight particle reorganisation as indicated by the increased particle number at these conditions (Fig. 2a). Liposomes are well-accepted standard nanoparticles in biomedical research, in our hands they overall performed comparable to EVs when loaded with a biologically relevant model enzyme.

**Figure 3:**
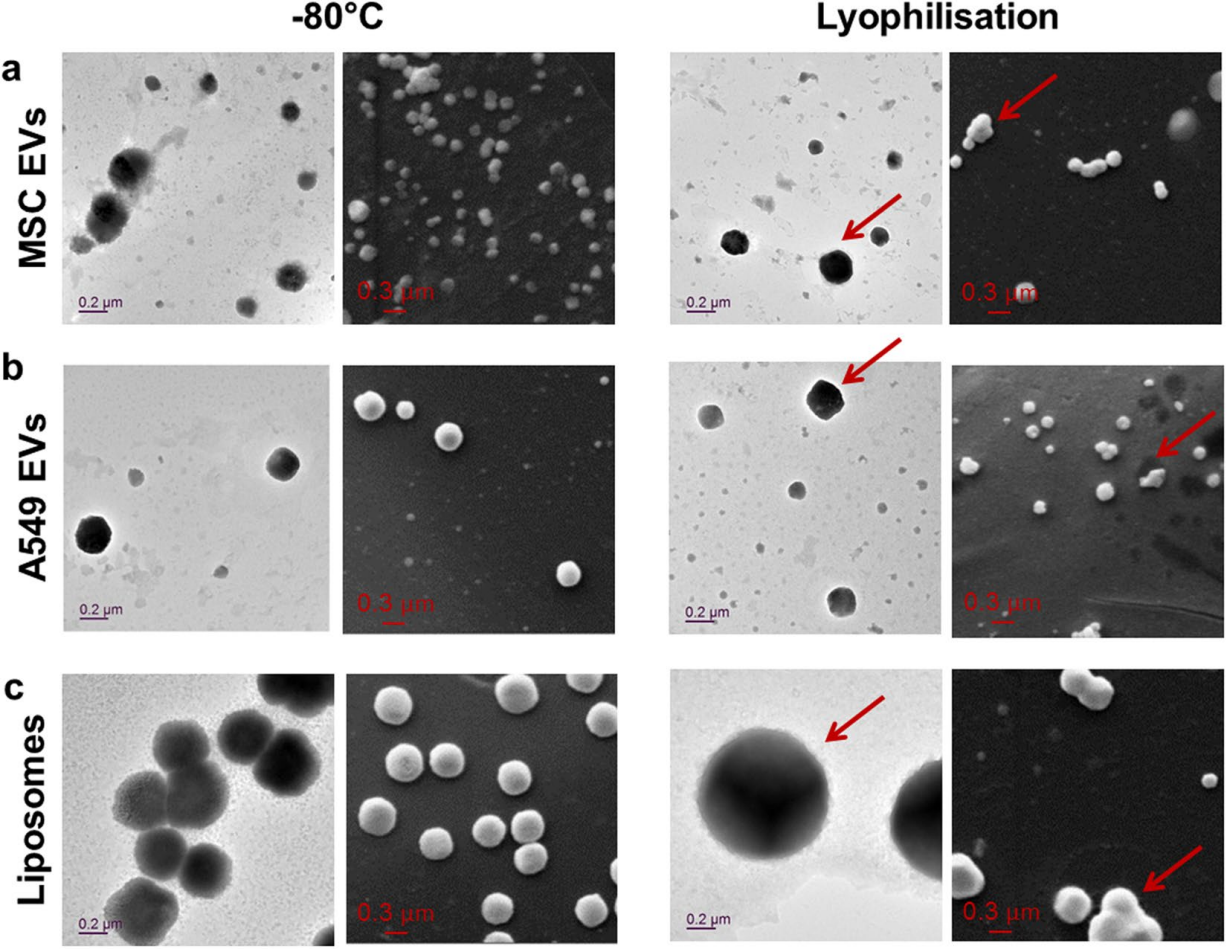
Morphology of glucuronidase-loaded EVs after lyophilisation. Representative electron micrographs of glucuronidase-loaded EVs from (**a**) MSC and (**b**) A549 cells, and (**c**) liposomes after 14 d storage at −80 °C or after lyophilisation. For each condition, transmission electron microscopy (TEM) upon phosphotungstic acid stain (left) and scanning electron microscopy (SEM) imaging of the same sample (right) was performed. Arrows in TEM and SEM images indicate presence of larger EVs and morphological changes in EV appearance (i.e., fusion or aggregation), respectively.

We subsequently performed electron microscopy analysis of selected samples, *i*.*e*., most and least stable EVs from MSC and A549, respectively, and liposomes (Fig. 3). Both transmission and scanning electron microscopy (TEM and SEM) confirmed that size distribution of MSC (Fig. 3a) and A549 EVs (Fig. 3b) remained relatively stable upon storage at −80 °C. Lyophilisation of these EVs induced a broadening of size distribution with creation of a few aggregates that may influence the biological activity of enzyme-loaded EVs (arrows in Fig. 3). Nevertheless, spherical shape and morphology of EVs did not appear to be substantially impaired during freeze-drying. Interestingly, lyophilisation of liposomes induced a strong increase in size compared to −80 °C (Fig. 3c) which was not observed as pronounced as in the bulk size measurements (Fig. 2a).

We then assessed the impact of various cryoprotecting agents on the stability and size of EVs. MSC EVs were selected due to their biomedical relevance in ongoing preclinical studies and for regenerative medicine applications^31,32^ and were lyophilised upon addition of different amounts of trehalose, mannitol or polyethylene glycol (PEG, Mw 400, SI Fig. 5). These compounds are commonly used for cryoprotection of nanoparticle-based formulations as they prevent ice crystal formation and allow improved dispersion when samples are rehydrated^33^. Both sugars efficiently improved EV stability during lyophilisation when added at 0.5–4% (*w/v*). Samples with mannitol concentrations above 4% (*w/v*) showed increasing particle concentration possibly due to sugar crystal formation because of limited solubility at higher concentrations. PEG induced EV aggregation at all concentrations tested which may be due to cross-linking of particles. Short PEGs have been shown in literature to induce steric stabilisation of liposomal formulations^34^, but in our hands this polymer seemed to interact with the EV surface (proteins) preventing its use as cryoprotectant for natural vesicles.

**Figure 4:**
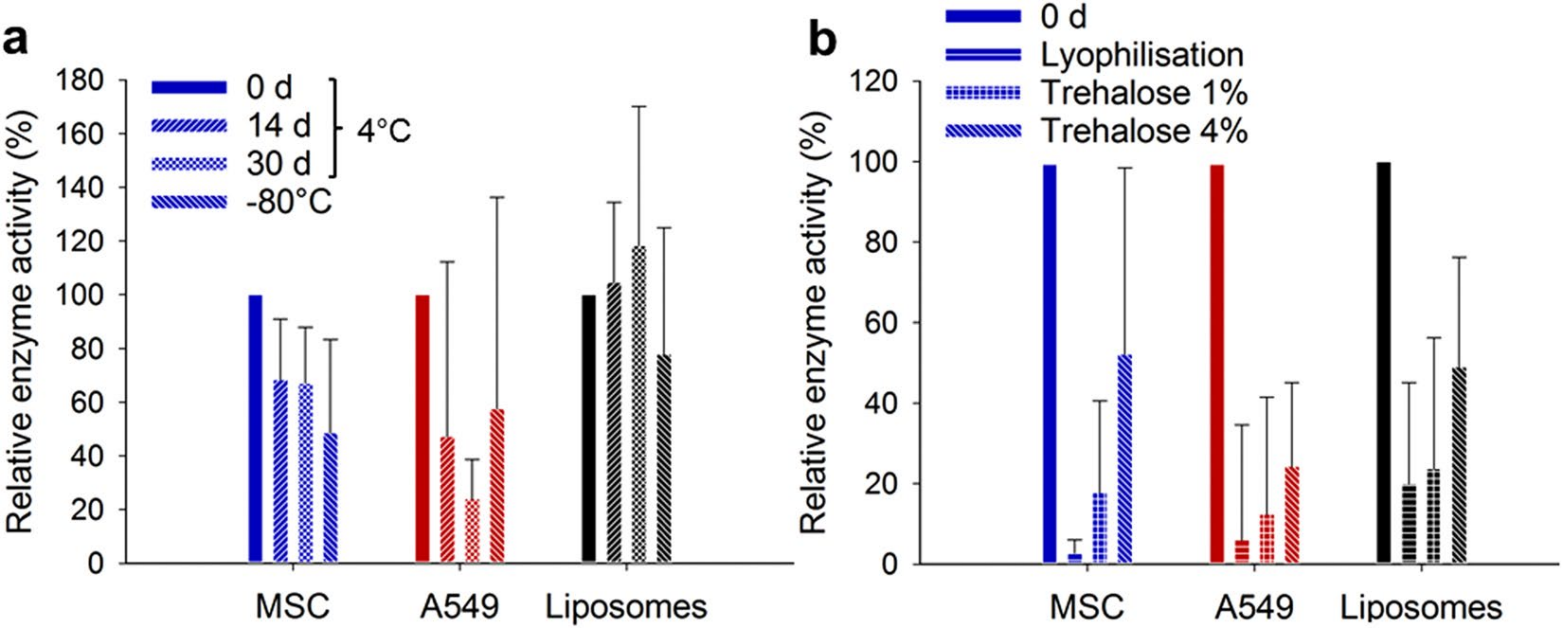
Cryoprotectants enhance functionality of enzyme-loaded EVs. The model enzyme glucuronidase was loaded into different EVs or liposomes. (**a**) Enzymatic activity of EVs from MSC, and A549 cells, or loaded liposomes was assessed after storage for 14 or 30 d at 4 °C, or for 30 d at −80 °C using fluorescein β-D-glucuronide as substrate. (**b**) Subsequently, the effect of cryoprotecting trehalose sugar on long-term stability and enzyme activity of EVs loaded with glucuronidase was studied. Samples were lyophilised with 1 and 4% (*w/v*) trehalose and stored for 14 d, and their enzyme activity was compared to the activity at day 0. Mean ± SD, *n* = 4–7.

We finally assessed the EV functionality upon lyophilisation selecting again MSC and A549 EVs as a more stable and a less stable EV type, and compared them with liposomes. All particles were loaded with glucuronidase to create vesicles whose enzyme activity was assessed after they were stored for up to 30 days at 4 °C or −80 °C. Figure 4a shows that all EVs exhibited a loss in enzymatic activity after one month of storage in the fridge, an effect most pronounced for A549 EVs. Storage at −80 °C circumvented this effect only to a certain extend. Liposomes appeared to be more stable but their enzymatic activity was also decreased during 30 days. For both natural and synthetic vesicles, lyophilisation without additives clearly reduced their bioactivity but addition of trehalose during freeze-drying substantially inverted this effect (Fig. 4b). Indeed, increasing amounts of cryoprotecting sugars directly correlated with the extent of activity recovery reaching enzyme activity values comparable to storage at −80 °C for MSC EVs. Our results indicate an important role of cryoprotectants during lyophilisation of EVs.

**Figure 5:**
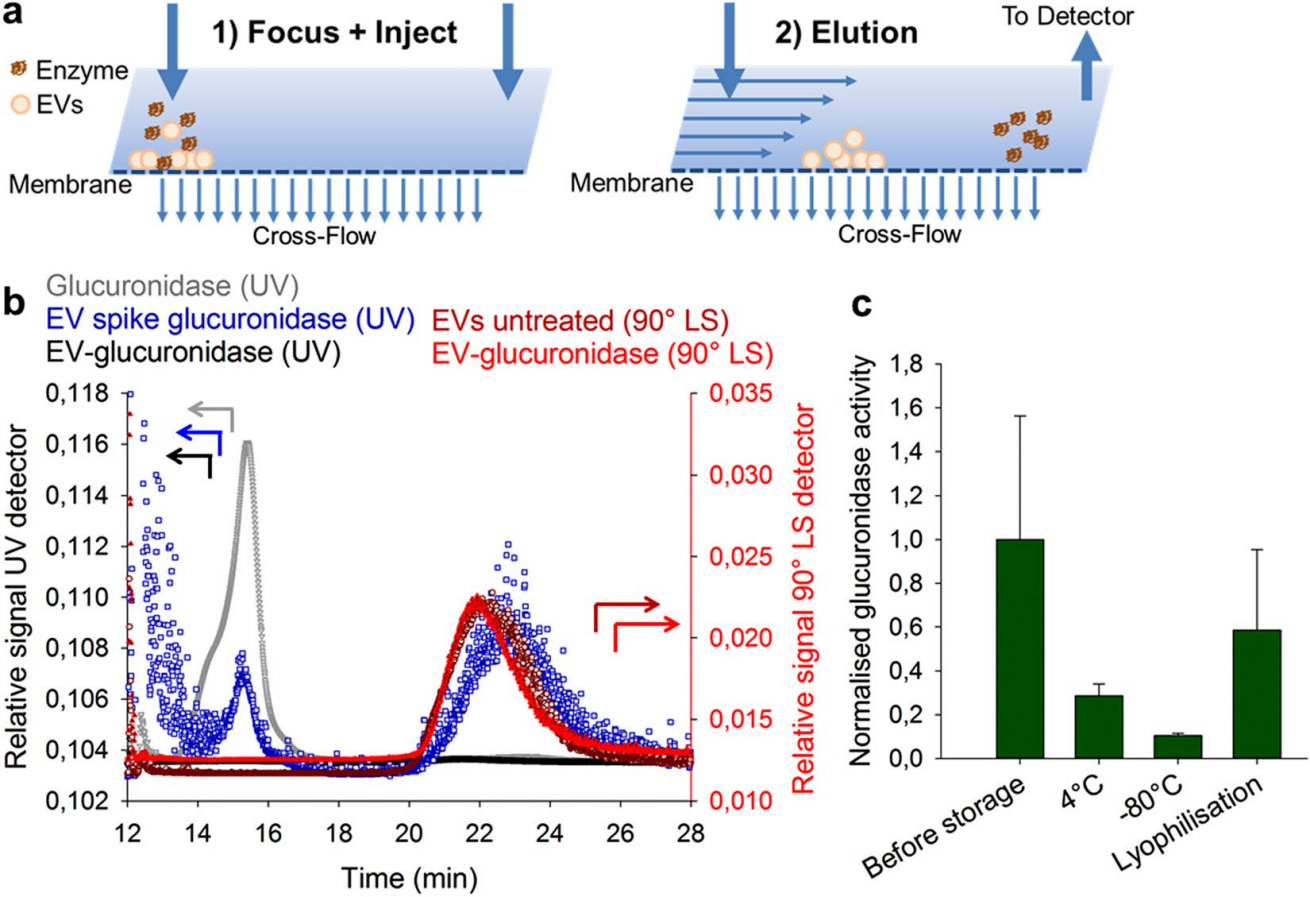
Analysis of glucuronidase-loaded EVs by asymmetric flow field-flow fractionation (AF4). (**a**) The working principle of AF4 consists of an injection step with a simultaneous sample focussing. Elution from the flow channel combined with a tangential cross-flow allows separation of particles and compounds by size. (**b**) Representative chromatograms of injections of free enzyme (glucuronidase 0.5 mg/mL), unmodified EVs, EVs spiked with glucuronidase (0.05 mg/mL), and EV-glucuronidase loaded samples (freshly purified by SEC). Detection of glucuronidase and EVs was conducted by UV spectroscopy and light scattering at 90° (90° LS), respectively. Smaller enzyme molecules are eluting earlier (~15 min) and larger EVs later (~22 min). Free glucuronidase cannot be LS-detected due to low scattering intensity. (**c**) Glucuronidase-loaded EVs isolated from HUVEC cells were stored for 7 days at 4 °C, −80 °C and lyophilised with 4% (*w/v*) trehalose. Their enzymatic activity was assessed after purification by AF4 and normalised to the average activity before storage. For EVs analysis, only the peak centre (*i*.*e*., 21–22 min) was collected by AF4 and enzyme activity was measured using fluorescein β-D-glucuronide. Mean ± SD, *n* = 3.

We further aimed at evaluating whether glucuronidase was stably encapsulated into EVs or whether it was loosely associated to vesicles. For this, AF4 was applied as a mild technique to segregate particles by applying a liquid cross-flow in a channel in contrast to the stationary phase in common chromatography methods^35^. AF4 was recently employed to distinguish functional subpopulations of EVs^36^ but has not been used to separate and analyse compounds loaded into EVs. As seen from Fig. 5a, in AF4 glucuronidase-loaded EVs were injected in a first focussing step followed by an elution step with an applied crossflow to separate any loosely attached or unbound glucuronidase (enzyme) from EVs. EVs from HUVEC cells were identified by light scattering detection and their chromatography peak clearly separated from the enzyme signal measured using the UV detector (Fig. 5b). When assessing the enzymatic activity of glucuronidase-loaded EVs before and after different storage conditions, we saw a decrease in activity for 4 °C and −80 °C which was less pronounced for EV samples lyophilised with 4% trehalose. Our data indicate that, EVs show inherent stability during lyophilisation and in our hands were better at protecting a model enzyme from degradation than storage at −80 °C. Their physicochemical properties are thus comparable or superior to synthetic liposomal analogues possibly opening further biological exploitations of EVs in the future. Moreover, AF4 is a viable alternative for separation of EVs from any undesired compounds as it causes less shear stress to the sample and allows efficient purification of particles in an automated manner.

Here, we show for the first time that EV functionality can be preserved using a simple and accessible method and we provide important evidence on EV handling during biological and medical evaluation. Lyophilisation was recently suggested as important alternative for storing EVs without compromising their biological cargoes^37^ but a detailed study of various EVs and the impact of freeze-drying was lacking to date. We could show that the particulate properties of EVs were not strongly influenced by lyophilisation, but we observed formation of few aggregates during direct lyophilisation of EVs. Such aggregates may influence sizing and detection of protein composition^15^ and they may impact on EV interactions studied in individual cell assays. Assessing the stability of enzymes upon encapsulation into EVs will additionally impact on extended investigation for regenerative medicine avenues where therapeutic enzymes may be combined with inherent tissue regenerative properties of EVs^38^ to render synergistic effects. Encapsulation of enzymes into EVs may also be a viable strategy for protecting these biomolecules upon systemic administration during enzyme-replacement treatment of metabolic or genetic dispositions^39^, as it was shown for Parkinson’s disease^40^. Finally, understanding EVs’ behaviour upon lyophilisation will significantly contribute to their pharmaceutical development as an off-the-shelf product with preserved bioactivity that does not require expensive cold chains or other sophisticated storage conditions. To this end, more detailed evaluations of EV-contained nucleic acids, such as miRNA, and the impact of lyophilisation on their stability need to be conducted in the future. EV-RNA is not only important for conveyance of cell responses but also in application of EVs in diagnostic settings. It would also be important to assess more detailed how lyophilised EVs interact with their target tissue^18^. Our findings have a broad importance for general EV applications in material sciences, engineering and bio-related sciences such as drug delivery, and they will enhance EV-related research by exploiting these endogenous carriers in their physiological state.

## Methods

### EV and liposome preparation and loading

Additional information on EV isolation and purification, and loading can be found in the Supplementary Information. Briefly, human mesenchymal stem cells (MSC, passages 3–8, MSCGM medium with bullet kit, Lonza), human umbilical vein endothelial cells (HUVEC, passages 3–8, EGM-2 medium with bullet kit, Lonza) and A549 lung cancer cells (A549, RPMI 1640 medium with 10% FCS) were cultured as described previously^22,41^. They were incubated under serum-free conditions for 48–72 h and at the following cell numbers: MSC 4 × 10^6^ cells/50 mL medium, HUVEC 10 × 10^6^ cells/50 mL medium, A549 16 × 10^6^ cells/50 mL medium (Fig. S1). 50 mL conditioned media were used to obtain one EV preparation and were subjected to differential centrifugation followed by a final ultracentrifugation step (120,000 × *g*, 2 h, 4 °C) on an Optima L-90 K (Beckman Coulter, Germany) to pellet EVs. EVs were loaded by incubation with 1.5 mg/ml β-glucuronidase and 0.1 mg/ml saponin (both Sigma, Germany) for 10 min at RT as reported previously^28^. Liposomes were prepared from DMPC and DPPC (5 mg/mL final concentration, molar ratio 2:3) using a lipid-film hydration method with 10 × extrusion through a polycarbonate membrane at 39 °C. For loading, 1.5 mg/ml β-glucuronidase was added to the re-hydration buffer.

Loaded and native EVs and liposomes were purified from residual protein aggregates and free enzyme using a sepharose CL-2B (Sigma, Germany) column of 17 mL volume and filtered PBS as eluent. Vesicles typically eluted in fractions 5–7 mL and were assessed using nanoparticle tracking analysis (Nanosight LM-10, Malvern Instruments, UK) equipped with a green laser measurement cell. Videos of 60 s were recorded using a camera level of 14–15 and detection threshold 10 (NTA 3.1 Software) to allow comparison between samples.

### Storage and analyses of EVs and liposomes

EVs or liposomes (200 µL) were stored at −80 °C, 4 °C, RT or upon lyophilisation. Lyophilisation was performed on an Alpha 2–4 freeze-dryer (Martin Christ, Germany) overnight and without addition of additives, or with supplementation of 0.5–4% (*w/v*) trehalose, 0.5–5% (*w/v*) mannitol, or 5–20% (*w/v*) polyethylene glycol (Mw 400 Da) (all from Sigma, Germany). For analysis, lyophilised samples were carefully rehydrated in milliQ water and vortexed for 30 s to ensure complete re-dispersion. All samples (−80 °C, 4 °C, RT or lyophilisation) were diluted to 200 µL and their size and yield was analysed by nanoparticle tracking analysis as described above. For each measurement, particle concentration was normalised to samples stored at −80 °C.

For TEM microscopy, EVs were applied to carbon-coated copper grids and fixed with 4% paraformaldehyde. Samples were washed with water, stained with 1% phosphotungstic acid for 20 s and dried overnight. TEM images were taken using a JEOL JEM 2011 (Oxford Instruments, UK). Subsequently, grids were fixed onto carbon disc covered SEM stubs and sputter-coated with gold. They were analysed using a EVO HD 15 (Zeiss, Germany).

Enzymatic activity of glucuronidase-loaded EVs and liposomes was assessed using fluorescein D-glucuronide (F2915, Thermo Fisher, USA) at 8.3 μg/mL final concentration (in 150 µL total reaction volume). Samples were diluted to 50 × 10^6^ particles/mL and incubated for 18 h at 37 °C and fluorescein release was measured at 516 nm (excitation 480 nm) on a M 200 plate reader (Tecan, Germany). Enzyme activity was normalised to the initial value at day 0.

### Asymmetric flow field-flow fractionation analysis

EV were separated from unencapsulated β-glucuronidase by asymmetric flow field-flow fractionation (AF4) on an Eclipse Dualtec system (Wyatt Technology Europe, Germany) connected to an isocratic pump, an online vacuum degasser and an autosampler (all Ultimate 3000 Dionex, Thermo Fisher Scientific, MA). AF4 was performed in a channel with a wide spacer of 350 µm thickness and a nadir cellulose membrane (30 kD molecular weight cut-off) set at 22 °C in a column oven (Hitachi High-Technologies Europe, Germany). The mobile phase, 150 mM phosphate buffered saline, pH 7.45 (Gibco PBS tablets, Thermo Fisher Scientific) was freshly filtered through a 0.1 µm Durapore PVDF membrane filter (Merck Chemicals GmbH, Germany). Between the HPLC pump and the AF4 channel an additional filter with a pore size of 0.1 μm was placed (PEEK Inline Filter Holder, Wyatt). The fractionated particles were detected with a UV detector at 280 nm (Thermo Fisher Scientific), and a multi-angle light scattering (MALS) detector (DAWN HELEOS II, Wyatt Technology Europe), with the laser set to 658 nm, calibrated using toluene, and normalized with bovine serum albumin protein as an isotropic scatterer standard. After a prefocusing step for 1 min with a focus flow of 1 mL/min, 300 µL of sample was injected at 0.2 mL/min for 10 min. After injection, the sample was eluted with a cross-flow of 2 mL/min decreasing to 0.1 mL/min in 8 min, followed by another 10 min of elution without cross-flow. Fractions were collected at 60 s intervals from 12.5 min to 27 min using an automated fraction collector (Thermo Fisher Scientific).

### Statistical analyses

All data are displayed as mean ± standard deviation and with *n* number of biological replicates performed. One-way analysis of variance followed by Dunnett’s *post-hoc* test was used for pairwise comparison with −80 °C control (SigmaPlot). Differences of *p* < 0.05 were considered significant.

## Electronic supplementary material

Supplementary Information

## Data Availability

The datasets generated during and/or analysed during the current study are available from the corresponding author on reasonable request.
