# Author Correction: Extracellular vesicles protect glucuronidase model enzymes during freeze-drying

**DOI:** 10.1038/s41598-019-48221-1

**Published:** 2019-10-25

**Authors:** Julia Frank, Maximilian Richter, Chiara de Rossi, Claus-Michael Lehr, Kathrin Fuhrmann, Gregor Fuhrmann

**Affiliations:** 1 Helmholtz Institute for Pharmaceutical Research Saarland (HIPS), Helmholtz Centre for Infection Research (HZI), Biogenic Nanotherapeutics group (BION), Campus E8.1, 66123 Saarbrücken, Germany; 2 Helmholtz Institute for Pharmaceutical Research Saarland (HIPS), Helmholtz Centre for Infection Research (HZI), Department of Drug Delivery (DDEL), Campus E8.1, 66123 Saarbrücken, Germany; 3 Department of Pharmacy, Saarland University, Campus Building E8.1, 66123 Saarbrücken, Germany

Correction to: *Scientific Reports* 10.1038/s41598-018-30786-y, published online 17 August 2018

In Figure 5b, “EV Spike glucuronidase (UV)” should read “EV spike glucuronidase (90° LS)”. The correct Figure 5b appears below as Figure. 1.

**Figure 1:**
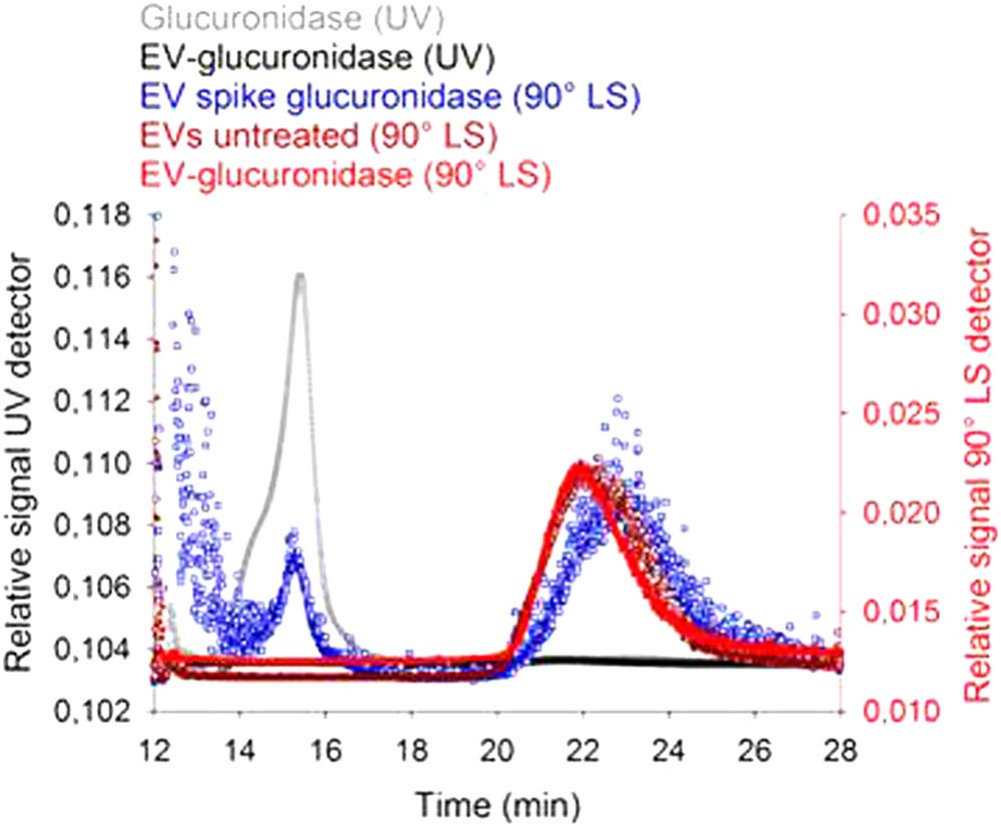
Analysis of glucuronidase-loaded EVs by asymmetric flow field-flow fractionation (AF4). (**a**) The working principle of AF4 consists of an injection step with a simultaneous sample focussing. Elution from the flow channel combined with a tangential cross-flow allows separation of particles and compounds by size. (**b**) Representative chromatograms of injections of free enzyme (glucuronidase 0.5 mg/mL), unmodified EVs, EVs spiked with glucuronidase (0.05 mg/mL), and EV-glucuronidase loaded samples (freshly purified by SEC). Detection of glucuronidase and EVs was conducted by UV spectroscopy and light scattering at 90° (90° LS), respectively. Smaller enzyme molecules are eluting earlier (~15 min) and larger EVs later (~22 min). Free glucuronidase cannot be LS-detected due to low scattering intensity. (**c**) Glucuronidase-loaded EVs isolated from HUVEC cells were stored for 7 days at 4 °C, −80 °C and lyophilised with 4% (*w/v*) trehalose. Their enzymatic activity was assessed after purification by AF4 and normalised to the average activity before storage. For EVs analysis, only the peak centre (*i.e*., 21–22 min) was collected by AF4 and enzyme activity was measured using fluorescein β-D-glucuronide. Mean ± SD, *n* = 3.

