# Supplementary material for: Extracellular vesicles protect glucuronidase model enzymes during freeze-drying
Source: Sci Rep. 2018 Aug 17;8:12377. doi: 10.1038/s41598-018-30786-y (PMC6098026; doi:10.1038/s41598-018-30786-y)
Supplement: Supplementary file 1 — Supplementary Information [file 41598_2018_30786_MOESM1_ESM.docx]

*Supplementary information*

**Extracellular vesicles protect glucuronidase model enzymes during freeze-drying**

Julia Frank ^a^, Maximilian Richter ^a^, Chiara de Rossi, Claus-Michael Lehr, Kathrin Fuhrmann and Gregor Fuhrmann*

^a^These authors contributed equally to the manuscript.

*Corresponding author, phone: +49 68198806 1500, email: gregor.fuhrmann@helmholtz-hzi.de

ORCID Gregor Fuhrmann: 0000-0002-6688-5126

**Contents:**

**Supplementary Methods**

**Figure S1.** **Cell morphology before and after conditioning in serum-free medium.**

**Figure S2. Size distribution of native and enzyme-laden liposomes and EVs.**

**Figure S3. Purification of glucuronidase-loaded EVs and liposomes by size-exclusion chromatography.**

**Figure S4. Long-term storage stability of enzyme-loaded liposomes and EVs.**

**Figure S5.** **Lyophilisation of EVs with different types and concentrations of cryoprotecting agents.**

**Supplementary Methods**

*EV isolation and loading*

Human mesenchymal stem cells (MSC, passages 3-8, MSCGM medium with bullet kit, Lonza), human umbilical vein endothelial cells (HUVEC, passages 3-8, EGM-2 medium with bullet kit, Lonza) and A549 lung cancer cells (A549, RPMI 1640 medium with 10% FCS) were cultured with 1% penicillin/streptomycin until near-confluent state. They were incubated with the respective serum-free medium for 48-72 h at the following cell numbers: MSC 4 × 10^6^ cells/50 mL medium, HUVEC 10 × 10^6^ cells/50 mL medium, A549 16 × 10^6^ cells/50 mL medium (**Fig. S1**). 50 mL conditioned media were used to prepare one EV preparation and centrifuged at 300 × g for 10 min to remove residual cells and at 3000 × g for 15 min to remove larger particles and cell debris. Ultracentrifugation of supernatants was performed using polyallomer tubes in a SW40 Ti or a SW32 Ti swing-out rotor and on a Beckman Optima L-90 K for 2 h at 120,000 × g and 4 °C. Supernatants were subsequently removed and pellets resuspended in typically 200 µL PBS to render concentrations of 10^10^ – 10^11^ particles / mL. Samples were stored in the fridge and used within 48 h because during this time frame EVs remained stable in size and concentration (**Fig. 1**).

For loading, typically 200 µL of EVs were mixed with 1.5 mg/ml β-glucuronidase (from *E. coli*, G7646) and 0.1 mg/ml saponin (47036) for 10 min at RT. EVs were purified from non-encapsulated, free enzyme using size exclusion chromatography (SEC) as described in the main text. All fractions were analysed by nanoparticle tracking analysis (particle count and size) and using a BCA assay (Pierce) according to the supplier’s instructions (protein analysis) (**Fig. S3**).

**
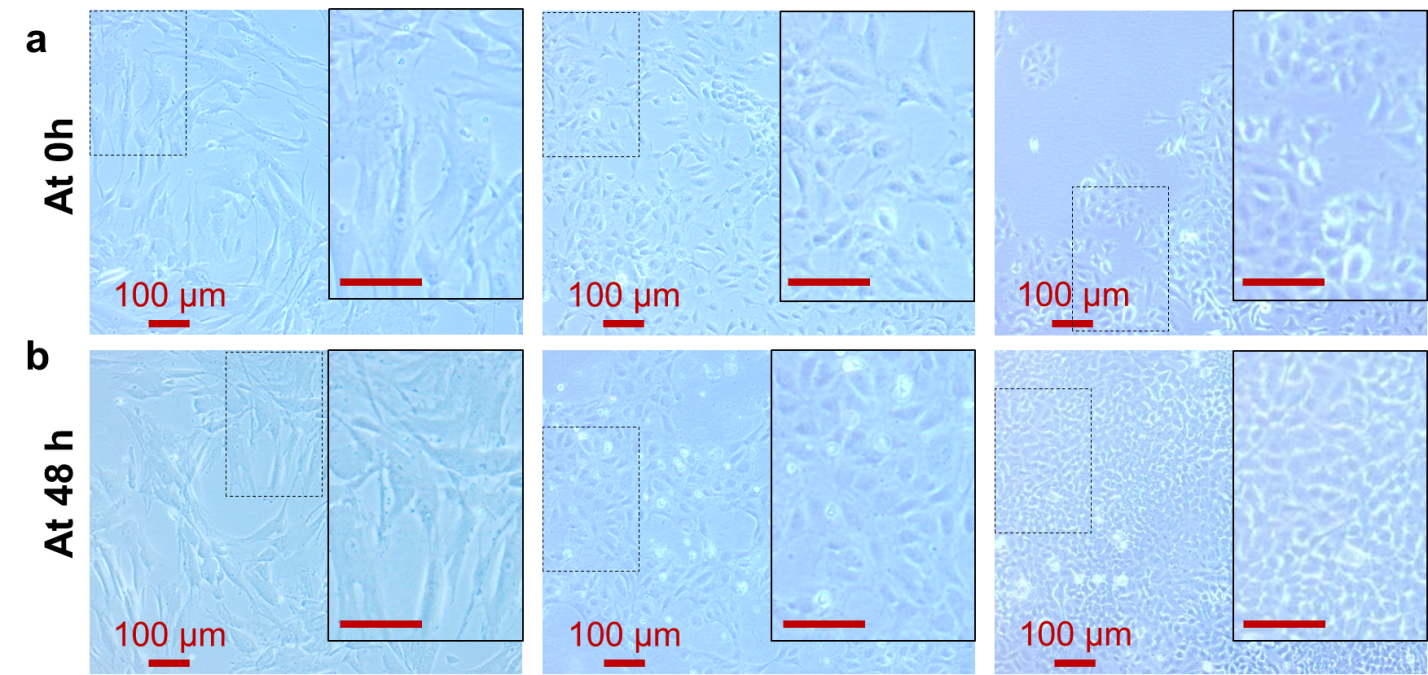
**

**Figure S1.** **Cell morphology before and after removal of conditioned medium.** MSC, HUVEC and A549 cells were cultured in their specific medium as indicated above. For EV isolation, they were conditioned with serum-free medium for 48 h and monitored (**a**) 0 h and (**b**) 48 h without observing any substantial changes in cell morphology and number. Images were taken at 10 × magnification. Insets were enlarged for better visualisation, scale bars of insets are 100 μm.

**
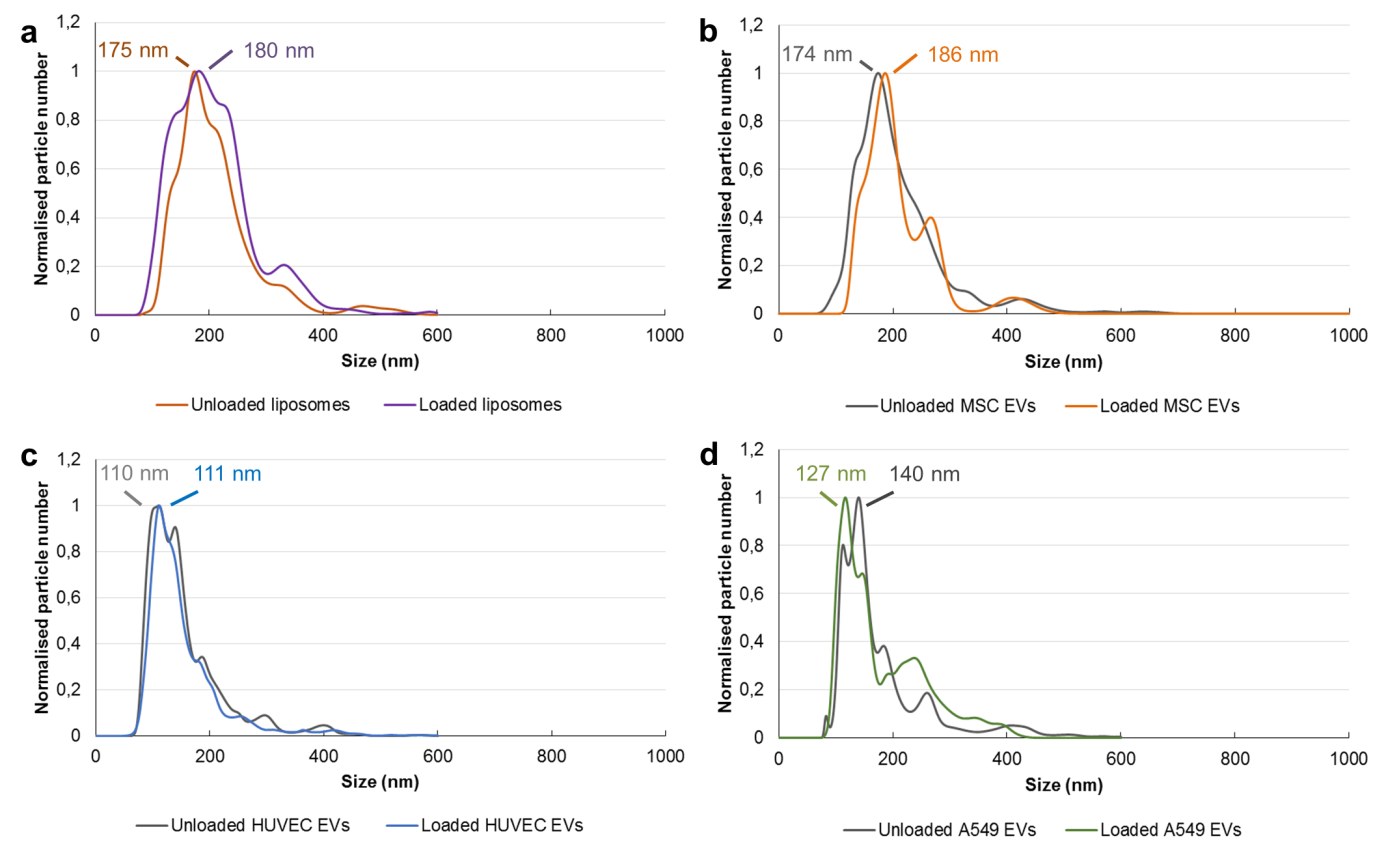
**

**Figure S2. Size distribution of native and enzyme-loaded liposomes and EVs.** (**a**) Liposomes, and EVs from (**b**) MSC, (**c**) HUVEC and (**d**) A549 cells were loaded with glucuronidase and their size distribution was compared to non-loaded liposomes, or native EVs using nanoparticle tracking analysis. In each panel, the size at the peak maximum is indicated.

**
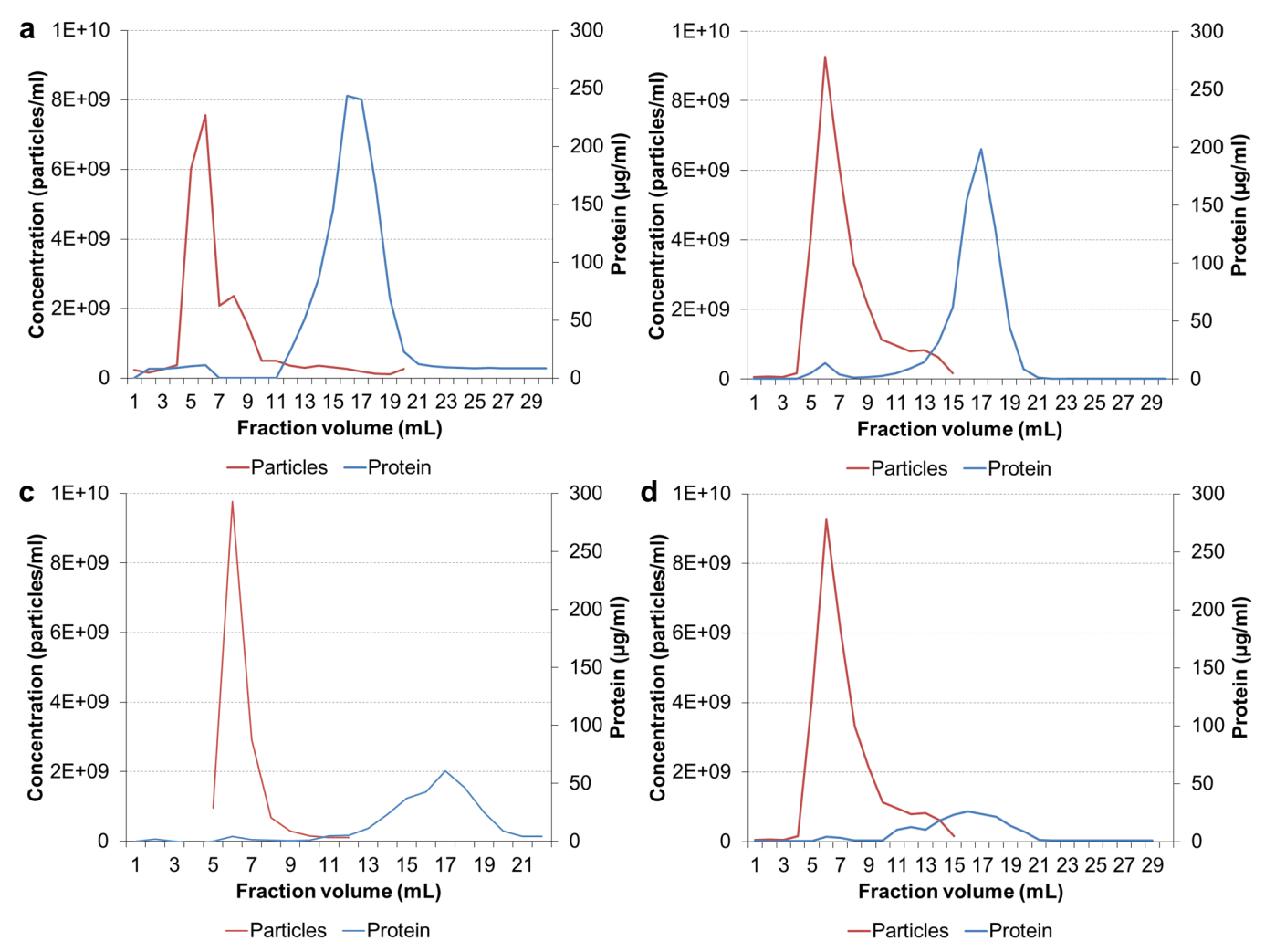
**

**Figure S3. Purification of glucuronidase-loaded EVs and liposomes by size-exclusion chromatography.** Representative elution profile and size of enzyme-loaded EVs from (**a**) MSC and (**b**) A549 cells, (**c**) HUVEC and (**d**) liposomes. Vesicles were loaded onto sepharose CL-2B columns of 17 mL and eluted using PBS. Their representative elution profile including particle count and protein content (determined by BCA assay) of all fractions is displayed.

**
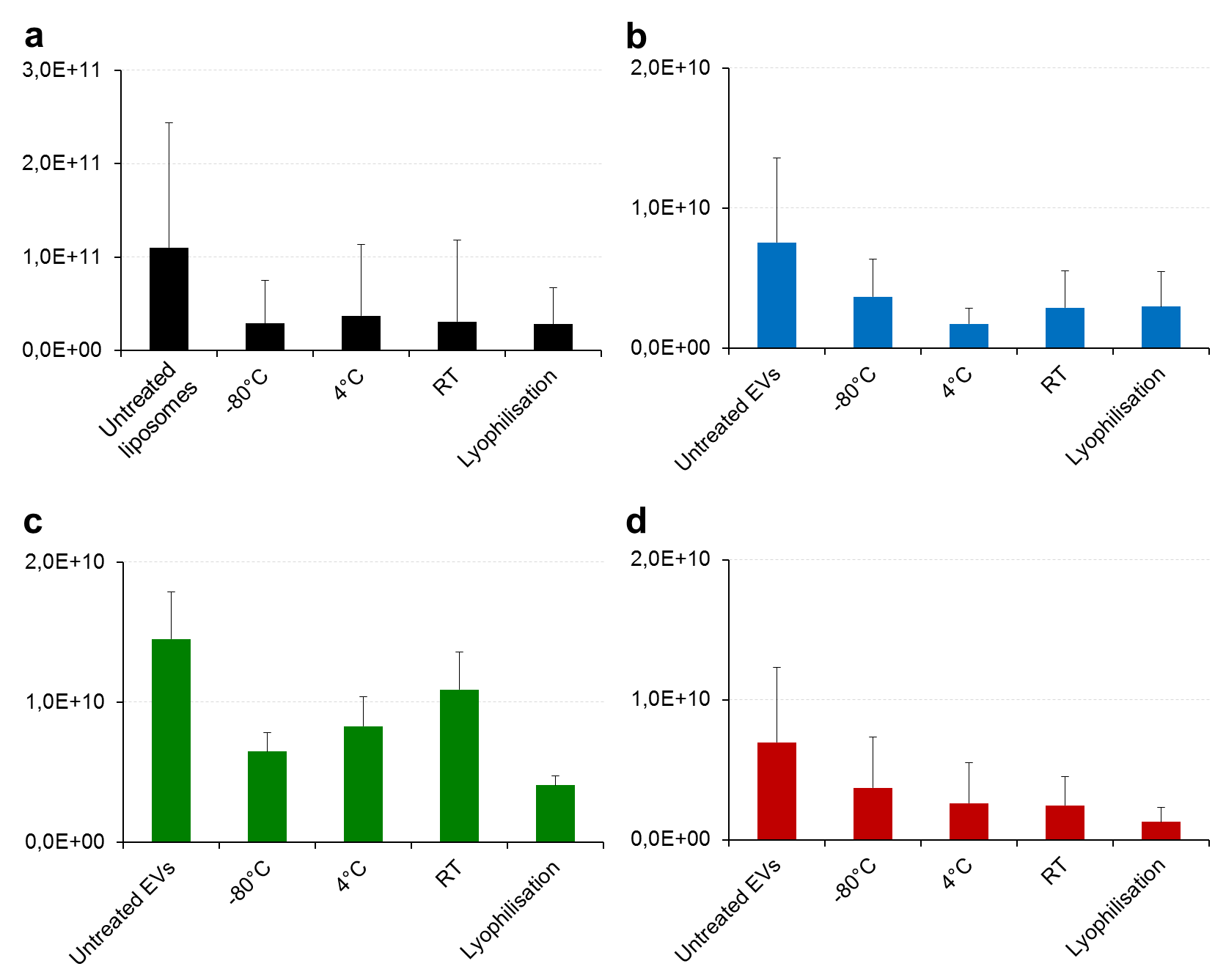
**

**Figure S4. Long-term storage stability of enzyme-loaded liposomes and EVs**. Particle concentration of (**a**) liposomes, and EVs from (**b**) MSC, (**c**) HUVEC and (**d**) A549 cells after loading of model enzyme glucuronidase was assessed. Natural and synthetic vesicles were stored for 14 d at various conditions (‑80 °C, 4 °C, RT, and lyophilisation) and analysed by nanoparticle tracking analysis in comparison to native EVs at day 0. Mean ± SD, *n* = 3.


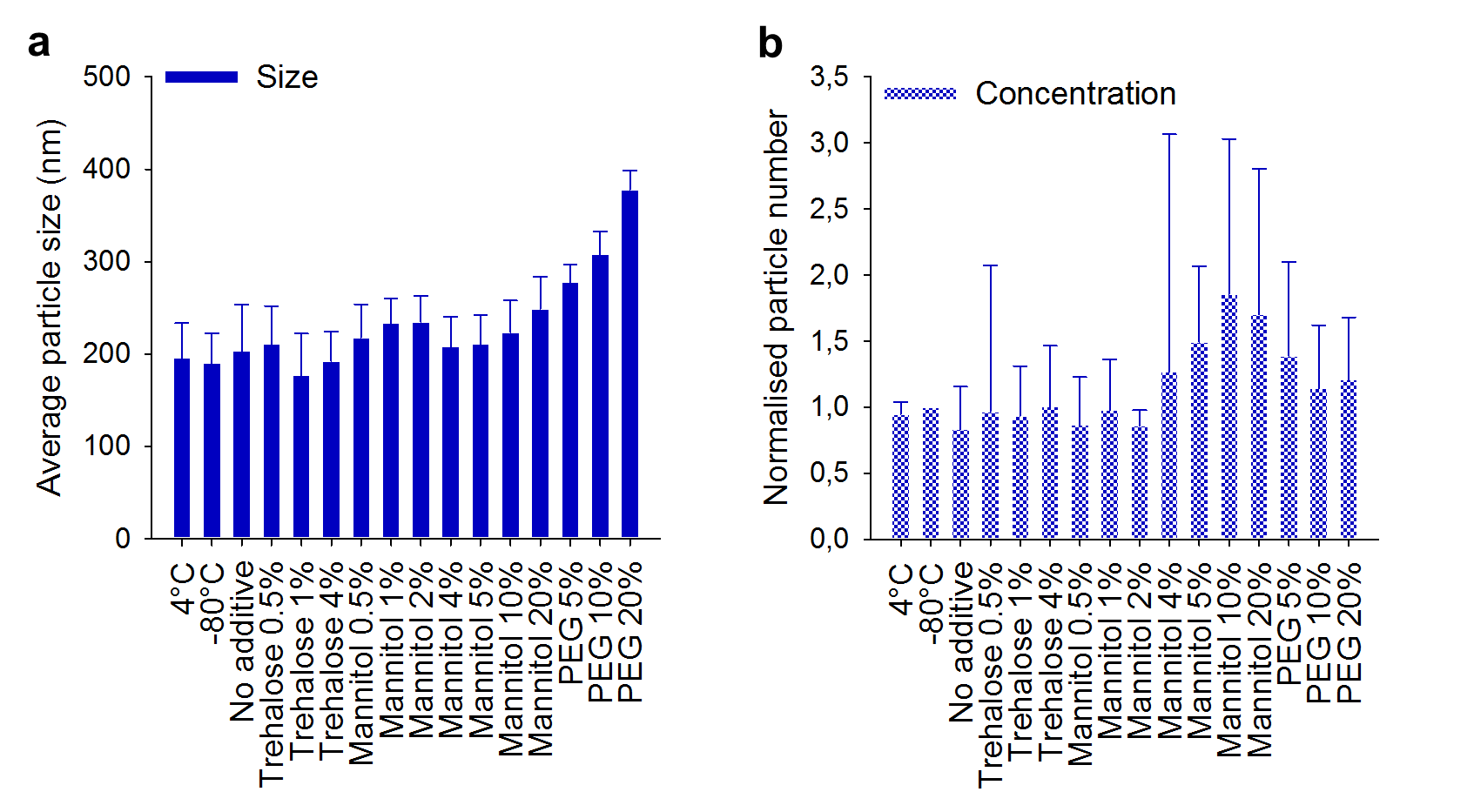
**Figure S5.** **Lyophilisation of EVs with different types and concentrations of cryoprotecting agents.** (**a**) Average size and (**b**) particle concentration of EVs from MSC cells stored at 4 °C, -80 °C, or freeze-dried without additives or upon addition of different concentrations of trehalose, mannitol, and polyethylene glycol (PEG, Mw 400 Da). Mean ± SD, *n* = 3–6.
